# Repurposing rupatadine as topical treatment against methicillin-resistant *Staphylococcus aureus*

**DOI:** 10.1186/s13568-025-01947-w

**Published:** 2025-09-23

**Authors:** Asmaa Farag, Dalia El-Damasy, Dalia Soliman, Galal Yahya, Kareem Ibrahim

**Affiliations:** 1 Department of Microbiology and Immunology, Faculty of Pharmacy, Egyptian Russian University, Cairo, Egypt; 2 Department of Pharmaceutical Chemistry, Faculty of Pharmacy, Al Azhar University, Cairo, Egypt; 3 Department of Microbiology and Immunology, Faculty of Pharmacy, Zagazig University, Zagazig, Egypt

**Keywords:** Aminoacyltransferase, Drug repurposing, FemA, Molecular docking, MRSA, Rupatadine

## Abstract

This study evaluates the potential of repurposed non-antibiotic drugs as possible treatments for methicillin-resistant *Staphylococcus aureus* (MRSA) skin infection, through a comprehensive approach combining in silico, in vitro, and in vivo methodologies. From 198 protein targets previously identified through multi-omics analysis, 10 essential, druggable, broad-spectrum targets were selected. Three promising non-antibiotic ligands (rupatadine, orlistat, and citric acid) were evaluated through minimum inhibitory concentration (MIC), minimum bactericidal concentration (MBC), molecular docking, cytotoxicity testing, and a murine model of MRSA skin infection. Rupatadine, orlistat, and citric acid showed MICs of 0.031 mg/mL, 1.5 mg/mL, and 2.5 mg/mL, respectively. Rupatadine demonstrated the most potent anti-MRSA activity, with molecular docking revealing favorable binding energy (− 3.45 kcal/mol) to Aminoacyl transferase (FemA). Serial passage experiments over eight passages showed no resistance development against rupatadine in one MRSA strain and only twofold increase in another, compared to 8–13 fold increases observed with conventional antibiotics. Checkerboard assays revealed synergistic effects between rupatadine and β-lactam antibiotics (cefazolin and cefotaxime, FICI = 0.3). FemA is essential in cell wall biosynthesis and represents an essential target for anti-MRSA agents. Functional enrichment and pathway network analyses revealed significant disruption of biological processes governing peptidoglycan biosynthesis and cell wall biogenesis, which indicates the possibility of rupatadine compromising *S. aureus* cell wall integrity through interference with peptidoglycan assembly pathways, supporting its potential as a repurposed anti-MRSA agent. Rupatadine showed favorable tolerance on human skin fibroblast cells (IC_50_: 1150 µg/mL), and significantly reduced bacterial count in a murine model.

**Supplementary Information:**

The online version contains supplementary material available at 10.1186/s13568-025-01947-w.

## Introduction

*Staphylococcus aureus* is a Gram-positive opportunistic pathogen that colonizes ~ 30% of healthy individuals but can cause diverse infections ranging from skin lesions to life-threatening conditions like endocarditis and pneumonia (Li et al. 2022; Parquet et al. 2018). This pathogen is the leading cause of skin infections globally, posing a significant public health concern when combined with antibiotic resistance crisis (Vena et al. 2023). Its clinical significance is heightened by extensive multidrug resistance, particularly against β-lactams, aminoglycosides, macrolides, and fluoroquinolones (Fuda et al. 2005; Hauschild et al. 2008; Kaatz and Seo 1997; Schmitz et al. 2000). Methicillin-Resistant *S. aureus* (MRSA) represents a particularly concerning "superbug" due to its broad antibiotic resistance profile and capacity to cause both community and healthcare-associated infections (Boswihi and Udo 2018; Nandhini et al. 2022). The World Health Organization has classified MRSA as a high-priority pathogen requiring urgent research and development of new antimicrobials, with an estimated 700,000 global deaths annually attributed to antimicrobial resistance (World Health Organization 2017).

The antibiotic resistance crisis has become an escalating concern, compounded by the fact that only few antibiotic agents have been approved by the Food and Drug Administration over the past two decades (Liu et al. 2021). The traditional drug discovery pipeline for new antibiotics requires 10–15 years and costs exceeding $1 billion, with high failure rates in clinical trials (DiMasi et al. 2016). In contrast, drug repurposing can reduce development timelines to 3–12 years with significantly lower costs and reduced risk profiles. This approach saves time, money, and resources compared to the conventional process of discovering and developing new antimicrobial agents (Ibrahim et al. 2020). Computational methods can be employed to achieve this goal through various in silico applications. While some studies have further validated putative drug targets in vitro, only a limited number have extended this validation to in vivo models (Ibrahim et al. 2023, 2020, 2022). This is a critical limitation, as computational predictions are not always accurate, with experimental evaluations typically confirming only 10–40% of the putative candidates as valid (Sadybekov and Katritch 2023).

Network pharmacology and systems biology approaches have emerged as powerful tools for identifying novel drug-target interactions and understanding complex mechanisms of action in antimicrobial drug discovery (Hopkins 2008). These computational frameworks can predict multi-target effects and optimize therapeutic outcomes while minimizing off-target toxicity.

Using multi-omics screening analysis, Rahman and Das identified several essential targets in MRSA, that were classified as either cytoplasmic or inner membrane proteins -which can serve as drug targets-; alongside extracellular proteins that hold potential as vaccine targets (Rahman and Das 2021).

In the present study, the findings of Rahman and Das were expanded by further validating the anti-MRSA activity of selected ligands. This validation was carried out through an integrative approach combining in silico, in vitro, and in vivo methodologies. This comprehensive approach bridges the gap between computational predictions and experimental validations, for the sake of combating antibiotic resistance crisis.

## Materials and methods

### Refinement of the initial protein targets

This study analyzed 198 MRSA protein targets from Rahman and Das (Rahman and Das 2021), initially filtering for druggable proteins with drug interaction potential. Using DrugBank (RRID:SCR_002700) (Wishart et al. 2018), information on potential binding ligands, including their status and classification was collected. We then assessed target presence across 54 common pathogens (Supplementary Table 1) using NCBI BLASTp (RRID:SCR_001010) (Altschul et al. 1997) with specific parameters (Expect threshold 1e^−5^, word size 5, BLOSUM62 matrix, gap costs 11/1), selecting only targets with scores > 200 in ≥ 80% of pathogens. Using the same parameters, protein targets were further compared against 16 common members of healthy human skin microbiome (Supplementary Table 2), in order to minimize potential disruption of beneficial skin flora (Byrd et al. 2018; Liu et al. 2025).

The three-dimensional (3D) structures of the MRSA protein targets shared across ≥ 80% of bacterial pathogens, were predicted using AlphaFold v2.3.2 computational modeling (RRID:SCR_025454) (Jumper et al. 2021). Each protein was identified by its corresponding UniProt ID (RRID:SCR_002380) (Consortium 2024). The predicted protein structures were subsequently visualized using Mol* Viewer to facilitate structural analysis and interpretation (Sehnal et al. 2021).

Finally, we eliminated targets lacking experimentally determined 3D structures in Protein Data Bank (PDB) (RRID:SCR_012820) (Berman et al. 2000), with strict criteria (E-value threshold 1e^−5^, sequence identity ≥ 90%) to ensure high-confidence structural matches.

### Filtering and selection of ligands

Following the refinement of the initial protein targets, a shortlist was generated, from which targets that only had antibiotic ligands were excluded. Among the remaining protein targets, the anti-MRSA activity of five potential binding ligands was evaluated through in vitro experimental assessment. The selected ligands covered a broad range of therapeutic applications:

- Vitamins: biotin (Bayer, Germany)
- Preservative: citric acid (Loba Chemie, India)
- Amino acid: glycine (Loba Chemie, India)
- Anti-hyperlipidemic agent: orlistat (Ocotber Pharma, Egypt)

A fifth ligand, rupatadine (Mash Premier, Egypt), was chosen based on structure-based virtual screening of the Staphylococcal Aminoacyltransferase (FemA) conducted on Rahman and Das’s study (Rahman and Das 2021; Wishart et al. 2018).

### Strains comparison to *Staphylococcus aureus* USA-300

Genetic relationships was assessed between our available test strains (*S. aureus* F-182 [ATCC 43300] (GenBank: AM980864.1), and *S. aureus *328 [ATCC 33591] (GenBank: CP077897.1)), and the USA-300 strain (GenBank: KY698019.1) used by Rahman and Das (Rahman and Das 2021). Comparisons used NCBI’s Needleman-Wunsch global alignment tool (RRID:SCR_004870) with default parameters (Reimer et al. 2022).

### Strain culturing and preservation

Bacterial strains were stored in trypticase soy broth (LabM, UK) with 20% glycerol at − 70 °C. Upon testing, strains were sub-cultured onto trypticase soy agar (LabM, UK) and incubated at 37 °C for 24 h under aerobic conditions. When selective growth of *S. aureus* was required, mannitol salt agar (MSA) (MAST, UK) was used (Missiakas and Schneewind 2013).

### Determination of minimum inhibitory concentrations (MIC)

The anti-MRSA activity of selected five ligands (biotin, citric acid, glycine, orlistat, and rupatadine) was evaluated against *S. aureus* strains F-182 and 328, using serial two-fold dilutions and broth microdilution method per CLSI M07-A9 guidelines (CLSI 2018). Vancomycin was included as a positive control for anti-MRSA activity (Brown et al. 2021). Sterile, flat-bottom 96-well microtiter plates (Corning, USA) were used for all assays. MIC was defined as the lowest concentration visibly inhibiting bacterial growth after 24 h of incubation at 37 °C. All tests were performed in triplicate, with growth control (bacteria + media), sterility control (media only), and solvent control (DMSO) included in each run.

#### Preparation of ligands solutions

Stock solutions were prepared by dissolving the compounds in sterile distilled water (Vancomycin, biotin, citric acid, and glycine) or 25% dimethyl sulfoxide (DMSO) (orlistat and rupatadine). The solutions were vortexed thoroughly and filtered through a 0.22 μm sterile filter (Sterilitech, USA).

#### Inoculum preparation

Direct colony suspension method was used to prepare inocula, according to CLSI M07-A9 guidelines (CLSI 2018). Colonies from fresh overnight cultures (24 h) on MSA plates were suspended in 0.9% saline and adjusted to 0.5 McFarland standard (approximately 1 × 10^8^ CFU/mL), diluted 1:20, and 10 µL was transferred to each well for a final concentration of 5 × 10^5^ CFU/mL.

### Determination of minimum bactericidal concentrations (MBC)

Minimum bactericidal concentrations (MBCs) of the most promising ligands were determined using broth microdilution method (Sykes and Rankin 2014). Following MIC experiments, 10 μL aliquots from clear wells (no visible growth) were sub-cultured onto MSA plates and incubated at 37 °C for 24 h. MBC was defined as the lowest concentration yielding no bacterial growth in the corresponding plates, with all tests performed in triplicate.

### Multiple sequence alignment and homology analysis

Based on MIC testing results, multiple sequence alignment of the putative protein targets for the most promising ligands (FemA and FabD targets of rupatadine and orlistat, respectively) from 12 MRSA strains (F-182, 328, USA300, USA300_TCH959, USA300_TCH1516, COL, KLT6, MRSA252, Mu50, MW2, N315, and NCTC8325) was performed using Clustal Omega (RRID:SCR_001591) (Madeira et al. 2022) to assess their conservation across different MRSA strains.

### Molecular docking of rupatadine to FemA protein target

Based on the relatively promising results of rupatadine, it was selected for further docking studies with its putative protein target (FemA), using Glide scoring function in Maestro 10.1 (RRID:SCR_016748); Schrödinger, 2015-Release-4).

#### Ligand preparation

LigPrep module was used for geometrical refining of chemical structures (Madhavi Sastry et al. 2013). Tautomers and conformations were generated using the Monte Carlo method in MacroModel (RRID:SCR_016747) version 9.8 (2010) (MacroModel 2010), with OPLS-2005 force field. Conformers were minimized using truncated Newton conjugate gradient minimization (Up to 500 iterations).

#### Protein preparation

The X-ray crystallographic structure of the *S. aureus* FemA protein (PDB ID: 1LRZ) was downloaded from the PDB. Protein preparation wizard of Maestro software was used. Structures were edited for missing hydrogens and proper bond orders. H-bonds were optimized, and non-hydrogen atoms were minimized to a default Root Mean Square Deviation value of 0.3.

#### Receptor grid generation

A receptor grid-box (90 Å × 90 Å × 90 Å) was generated with center at x = 42.075, y = 62.493, z = 90.597, and a grid space of 0.375.

#### Molecular docking analysis

Flexible docking was performed using the standard precision feature of Glide module, version 5.6 (2010) (Friesner et al. 2004).

### Target prediction, enrichment analysis, and network construction

To identify other potential molecular targets for the selected ligand, SwissTargetPrediction (RRID:SCR_023756) (Daina et al. 2019) and PharmMapper (RRID:SCR_022604) (Wang et al. 2017) were employed. Predicted targets were screened for relevance to microbial physiology and virulence, and cross-referenced with microbial protein entries in the UniProt database (Consortium 2024), to ensure specificity. Subsequently, the refined targets list was subjected to Gene Ontology (GO) and pathway enrichment analysis using the Database for Annotation, Visualization, and Integrated Discovery (DAVID) (RRID:SCR_001881) (Sherman et al. 2022) and Kyoto Encyclopedia for Genes and Genomes (KEGG) (RRID:SCR_012773) (Kanehisa and Sato 2020) databases. Enriched pathways with a false discovery rate < 0.05 were considered statistically significant.

To visualize the pharmacological landscape, protein–protein interaction networks were generated using ShinyGO v0.81 (RRID:SCR_019213) (Ge et al. 2020), which also supported additional GO analysis. The networks were analyzed to identify central nodes and key hubs, using degree centrality as a measure of network influence.

### In vitro cytotoxicity testing of rupatadine

Rupatadine cytotoxicity was assessed using Sulforhodamine B (SRB) colorimetric assay on human skin fibroblasts (HSFs) (AcceGen Biotech Cat. # ABC-TC3451, USA; Obtained in September 2023). Cells were maintained in Dulbecco’s Modified Eagle Medium with antibiotics and 10% fetal bovine serum in humidified 5% CO₂ at 37 °C.

Cell suspensions (5 × 10^3^ cells in 100 μL) were treated in triplicate with rupatadine (0.32–3200 μg/mL) and incubated for 2 h. Cells were fixed with 10% trichloroacetic acid for 1 h at 4 °C, washed, stained with 0.4% SRB for 10 min, washed with 1% acetic acid, and air-dried. After dissolving protein-bound SRB in 10 mM TRIS, absorbance was measured at 540 nm (Allam et al. 2018). DMSO (vehicle control) and Doxorubicin (positive control) effects were tested under identical conditions (Sadeghi-Aliabdai et al. 2010). Half Maximal Inhibitory Concentration (IC₅₀) was calculated as the concentration inhibiting cell viability by 50% (Mahavorasirikul et al. 2010).

### Serial passage resistance development assay

To evaluate the potential for resistance development, serial passage experiments were conducted for rupatadine, vancomycin, and ciprofloxacin against *S. aureus* strains F-182 and 328. Vancomycin was selected as one of the primary treatments for MRSA infections (Brown et al. 2021), while ciprofloxacin was used according to CLSI guideline for studying resistance evolution in organisms such as *S. aureus* and *Pseudomonas aeruginosa* (CLSI 2020)*.*

The assay was performed according to Haas et al., with modifications (Haas et al. 1990). Briefly, following MIC determination, a subculture on MSA was made from wells with sub-inhibitory concentrations (0.5 × MIC) of each compound. Colonies were then suspended in saline and adjusted to 0.5 McFarland standard (approximately 1 × 10^8^ CFU/mL), diluted 1:20, and 10 µL was transferred to each well for a final concentration of 5 × 10^5^ CFU/mL. The MIC was re-assessed using the new inoculum and determined every second passage. The fold-increase in MIC relative to baseline (passage 0) was calculated and plotted against passage number. Experiments were performed in triplicate, and the process was continued for eight consecutive passages to monitor resistance development patterns over time.

### Evaluation of potential synergistic effects

Checkerboard assays were performed to assess potential synergistic interactions between rupatadine and selected antibiotics against MRSA strains F-182 and 328 (Bellio et al. 2021). The antibiotic panel included both resistance-prone agents (amikacin (Amoun, Egypt), cefazolin (Pharco, Egypt), cefotaxime (UP Pharma, Egypt)) (Lade and Kim 2023), and first-line anti-MRSA drugs (linezolid (Otuska, Egypt), vancomycin (Sigmatec, Egypt)) (Azzam et al. 2023). Drug interactions were quantified using the fractional inhibitory concentration index (FICI), calculated as follows:

$$ {\text{FICI}} = \frac{{{\text{MIC}}_{{{\text{A}}\left( {{\text{combination}}} \right)}} }}{{{\text{MIC}}_{{{\text{A}}\left( {{\text{alone}}} \right)}} }} + \frac{{{\text{MIC}}_{{{\text{B}}\left( {{\text{combination}}} \right)}} }}{{{\text{MIC}}_{{{\text{B}}\left( {{\text{alone}}} \right)}} }} $$

Synergistic effect was defined as FICI ≤ 0.5; additive effect was defined as 0.5 < FICI ≤ 1; indifference effect was defined as 1 < FICI ≤ 4; and antagonism effect was defined as FICI > 4 (Dupieux et al. 2017).

### Efficacy of rupatadine In vivo using murine MRSA skin infection model

The murine MRSA skin infection model was approved by Zagazig University’s IACUC (Approval no. ZU-IACUC/3/F/452/2023) following established guidelines (National Research Council 2010a; Salem et al. 2021).

Thirty-two male Swiss mice (RRID:IMSR_TAC:sw), 6–8 weeks old, 22 ± 2 gm weight, obtained from Modern Veterinary Office (Cairo) were housed in Makrolon cages (8 per cage) at 25 °C with 12 h light/dark cycle and ad libitum food and water. After one-week acclimation, mice were shaved on the posterior upper back, skin was sterilized with 70% alcohol, and any mice with wounds were excluded. Each mouse received a subcutaneous injection of 40µL *S. aureus* F-182 (5.5 × 10⁸ CFU in Phosphate Buffer Saline (PBS)), then were divided into four treatment groups: rupatadine, vancomycin, PBS, and 25% DMSO. Vancomycin was selected as one of the primary treatment for MRSA infections (Brown et al. 2021), 25% DMSO served as a vehicle control, and PBS as a negative control.

Both rupatadine and vancomycin were freshly prepared daily to achieve concentrations of 320 μg/ml and 10 μg/ml, respectively. From days 1–5 post-infection, each of the four groups was treated topically by pipetting 100 μL of the corresponding drug once daily on the shaved skin followed by gentle rubbing. On day 6, mice were euthanized. Prior to euthanization, anesthesia using 50 mg/kg intraperitoneal thiopental (EIPICO, Egypt) was administrated to ensure rapid unconsciousness before euthanization by cervical dislocation (Vogler 2006). A 1 cm × 1 cm skin section was then excised, weighed and homogenized in 1 mL PBS, followed by eight tenfold serial dilutions. An aliquot of 10 μL was then pipetted onto MSA plates in triplicate and incubated at 37 °C. After 24 h incubation, *S. aureus* colonies were counted, and the total count per gram of skin was calculated.

### Statistical analysis

GraphPad Prism (RRID:SCR_002798) version 8.4.2 was used to perform statistical analyses. Non-linear regression analysis and log (inhibitor) vs. normalized response—Variable slope equation was used to calculate IC_50_. One-way ANOVA was used to calculate the difference and significance among the four in vivo treatment groups.

## Results

### Refinement of initial protein targets

From the initial 198 MRSA protein targets identified by Rahman and Das [14], analysis revealed 53 druggable targets with 163 unique approved drugs as potential binding ligands. Of these drugs, 55.2% were investigational, 19% had veterinary approval, 9.8% were nutraceuticals, 7.4% were withdrawn, and 1.8% were experimental (Supplementary Table 3). Further screening for presence in common pathogens (Score > 200) yielded 21 druggable, cytoplasmic, non-human homologous targets present in ≥ 80% of common pathogens. These 21 targets were additionally screened against the 16 common healthy skin microbiome species (Table 1).

**Table 1:** Shortlisted MRSA protein targets present in ≥ 80% of common pathogens and their relative presence in healthy human skin microbiome

	UniProt ID	Protein name	Presence in common pathogens (%)	Presence in skin microbiome (%)
1	A8Z2L6	Acetyl-coenzyme A carboxylase carboxyl transferase subunit beta (AccD)	89	31.25
2	Q2G268	Coenzyme A biosynthesis bifunctional protein (CoaBC)	87	87.5
3	Q2FZY5	Cysteine desulfurase	87	87.5
4	Q93QD4	Malonyl CoA-acyl carrier protein transacylase (FabD)	81	31.25
5	A8Z088	Beta-ketoacyl-[acyl-carrier-protein] synthase III (FabH)	91	18.75
6	Q6GI75	Enoyl-[acyl-carrier-protein] reductase [NADPH] (FabI)	81	25
7	P0A040	Glutamine synthetase (GlnA)	93	93.75
8	A8Z4T2	Chaperonin GroEL	100	100
9^†^	Q5HJZ0 ^†^	DNA gyrase subunit A (GyrA)	98	100
10	Q2G078	Ribonucleoside-diphosphate reductase subunit alpha (NrdA)	85	75
11	Q2FYS4	DNA topoisomerase IV subunit A (ParC)	100	100
12	P0C1S7	DNA topoisomerase IV subunit B (ParE)	100	100
13	A8Z1R9	Phenylalanine-tRNA ligase subunit alpha (PheS)	96	100
14	Q2FHU2	Phenylalanine-tRNA ligase subunit beta (PheT)	96	100
15^†^	Q2FER5 ^†^	DNA-directed RNA polymerase subunit alpha (RpoA)	93	100
16^†^	A8YZP0 ^†^	DNA-directed RNA polymerase subunit beta (RpoB)	100	100
17^†^	Q2FEP5 ^†^	30S ribosomal protein S3 (RpsC)	98	93.75
18^†^	P0A0J0 ^†^	RNA polymerase sigma factor (SigA)	100	100
19	Q2G041	Thioredoxin reductase	98	100
20	Q2FY51	Dihydrolipoyl dehydrogenase	89	100
21	Q2FE05	UTP-glucose-1-phosphate uridylyltransferase (GtaB)	89	43.75

^†^Proteins targets with only antibiotic ligands

**Fig. 1:**
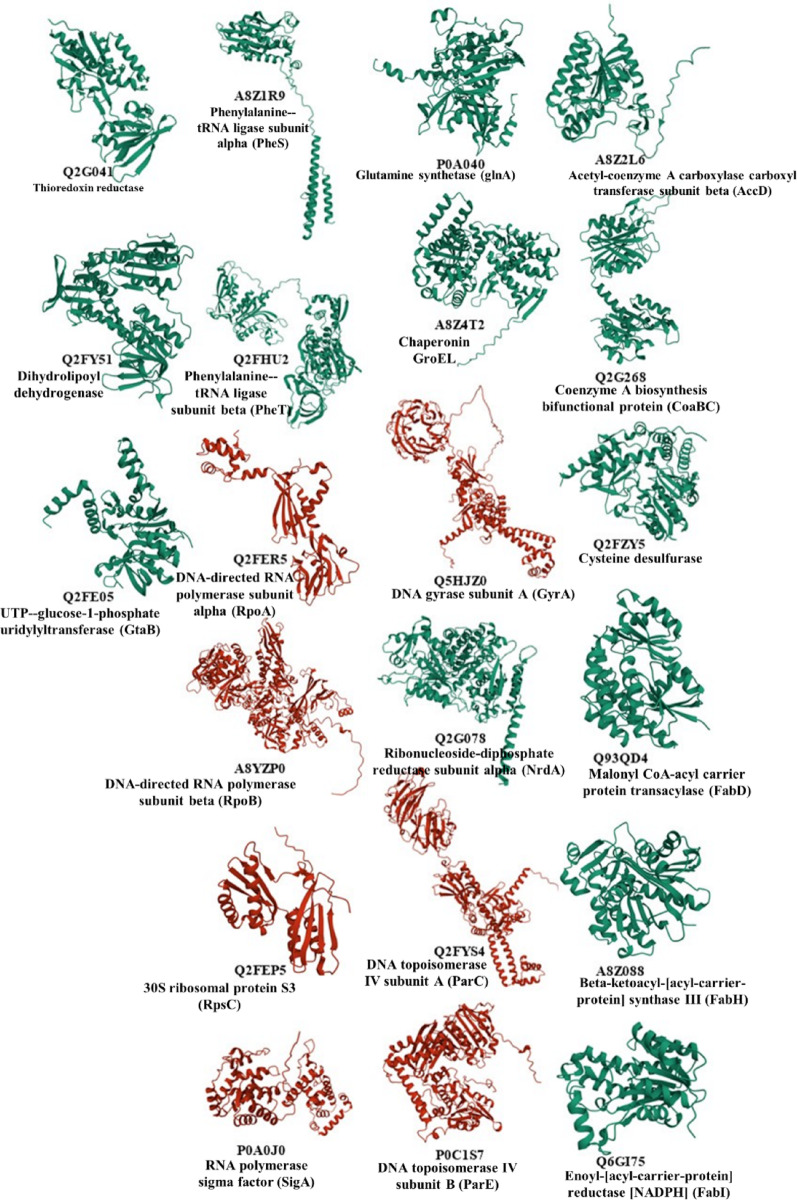
Predicted 3D Structure of 21 MRSA’s Protein Targets Shared in ≥ 80% of Bacterial Pathogens. Proteins were named by their UniProt ID, and were modelled with AlphaFold v2.3.2 to predict the 3D structure. The predicted 3D structure of each protein was visualized using Mol* Viewer. Proteins visualized in red color are either protein targets with antibiotic ligands or with identified 3D structure.

AlphaFold v2.3.2 successfully predicted the 3D structures of all 21 MRSA protein targets that are conserved across ≥ 80% of bacterial pathogens (Fig. 1). The predicted structures displayed high confidence scores, with the majority showing well-defined secondary structure elements and functional domains.

Out of the resulted targets, 10 proteins had available PDB crystallographic structures, after excluding six proteins lacking 3D structures and five with only antibiotic ligands (Table 2).

**Table 2:** Protein Data Bank (PDB) IDs of selected MRSA protein targets with available three-dimensional (3D) structures

	Protein name	PDB ID		
1	Acetyl-coenzyme A carboxylase carboxyl transferase subunit beta (AccD)	2F9I	5KDR	
2	Cysteine desulfurase	8D8S		
3	Malonyl CoA-acyl carrier protein transacylase (FabD)	3IM9		
4	Beta-ketoacyl-[acyl-carrier-protein] synthase III (FabH)	1ZOW	3IL7	6KVS
5	Enoyl-[acyl-carrier-protein] reductase [NADPH] (FabI)	3GNS	4BNG	4CV1
		3GNT	4BNH	4D41
		3GR6	4BNI	4D42
		4ALL	4BNJ	4D43
		4ALI	4BNK	4D44
		4ALJ	4BNL	4D45
		4ALK	4BNM	4FS3
		4ALM	4BNN	6YUR
		4ALN	4CUZ	6TBB
		4BNF	4CV0	6TBC
6	Glutamine synthetase (GlnA)	7TF6	7TF7	7TDV
7	DNA topoisomerase IV subunit A (ParC)	2INR		
8	DNA topoisomerase IV subunit B (ParE)	4URL	4URN	
9	Phenylalanine–tRNA ligase subunit alpha (PheS)	2RHQ	2RHS	
10	Thioredoxin reductase	4GCM		

### Filtering and selection of ligands

Ten targets yielded 19 potential binding ligands, with rupatadine added based on virtual screening of FemA protein, bringing the total to 20 potential ligands (Table 3).

**Table 3:** Shortlisted non-antibiotic anti-MRSA ligands: categories, names, status, and target proteins

Category	Name	Status	Target
Anti-histamine	Rupatadine	A	Aminoacyltransferase
Anti-hyperlipidemia	Orlistat	A, I	Malonyl CoA-acyl carrier protein transacylase
Anti-tumor	Etoposide	A	DNA topoisomerase IV subunit A
			DNA topoisomerase IV subunit B
	Amsacrine	A, I	DNA topoisomerase IV subunit B
	Valrubicin	A	
	Idarubicin	A	
Preservative	Citric acid	A, N, VA	Glutamine synthetase
	Lauric acid	A, E	3-oxoacyl-[acyl-carrier-protein] synthase3
	Triclocarban	A	Enoyl-[acyl-carrier-protein] reductase [NADPH]
	Triclosan	A, I	
Supplement/Amino acids	Cysteine	A, N	Cysteine desulfurase
	Glycine	A, N, VA	Thioredoxin reductase
	NADH	A, N	
	Phenylalanine	A, I, N	Phenylalanine-tRNA ligase subunit alpha
	Selenium	A, I, VA	Cysteine desulfurase
	Valine	A, N	Acetyl-coenzyme A carboxylase carboxyl transferase subunit beta
Vitamin/Vitamin derivatives	Biotin	A, I, N	
	Flavin adenine dinucleotide	A	Thioredoxin reductase
	Pyridoxal phosphate	A, I, N	Cysteine desulfurase
	Vitamin A	A, N, VA	Enoyl-[acyl-carrier-protein] reductase [NADPH]

A: Approved; E: Experimental; I: Investigational, N: Nutraceutical; VA: Vet Approved

Five ligands were then selected to represent broad range therapeutic applications, including biotin vitamin, citric acid preservative, glycine amino acid, the anti-hyperlipidemia agent: orlistat, and the anti-histamine agent: rupatadine.

### Anti-MRSA activity of the selected ligands

Both *S. aureus* F-182 and 328 strains to be used in testing in this study showed > 99% sequence homology with *S. aureus* USA-300 strain using NCBI’s Needleman-Wunsch alignment.

Biotin (125 mg/mL) and glycine (50 mg/mL) showed no anti-MRSA activity. The MICs for the remaining ligands were: citric acid (2.5 mg/mL), orlistat (1.5 mg/mL), and rupatadine (0.03125 mg/mL). Vancomycin demonstrated an MIC of 1 μg/mL against both MRSA strain, which falls within the CLSI susceptibility breakpoint (≤ 2 μg/mL) (CLSI 2020).

MBCs were determined for orlistat (1.5 mg/mL, equal to MIC) and rupatadine (0.0625 mg/mL, twice its MIC).

### Conservation of FemA and FabD proteins across multiple MRSA strains

**Fig. 2:**
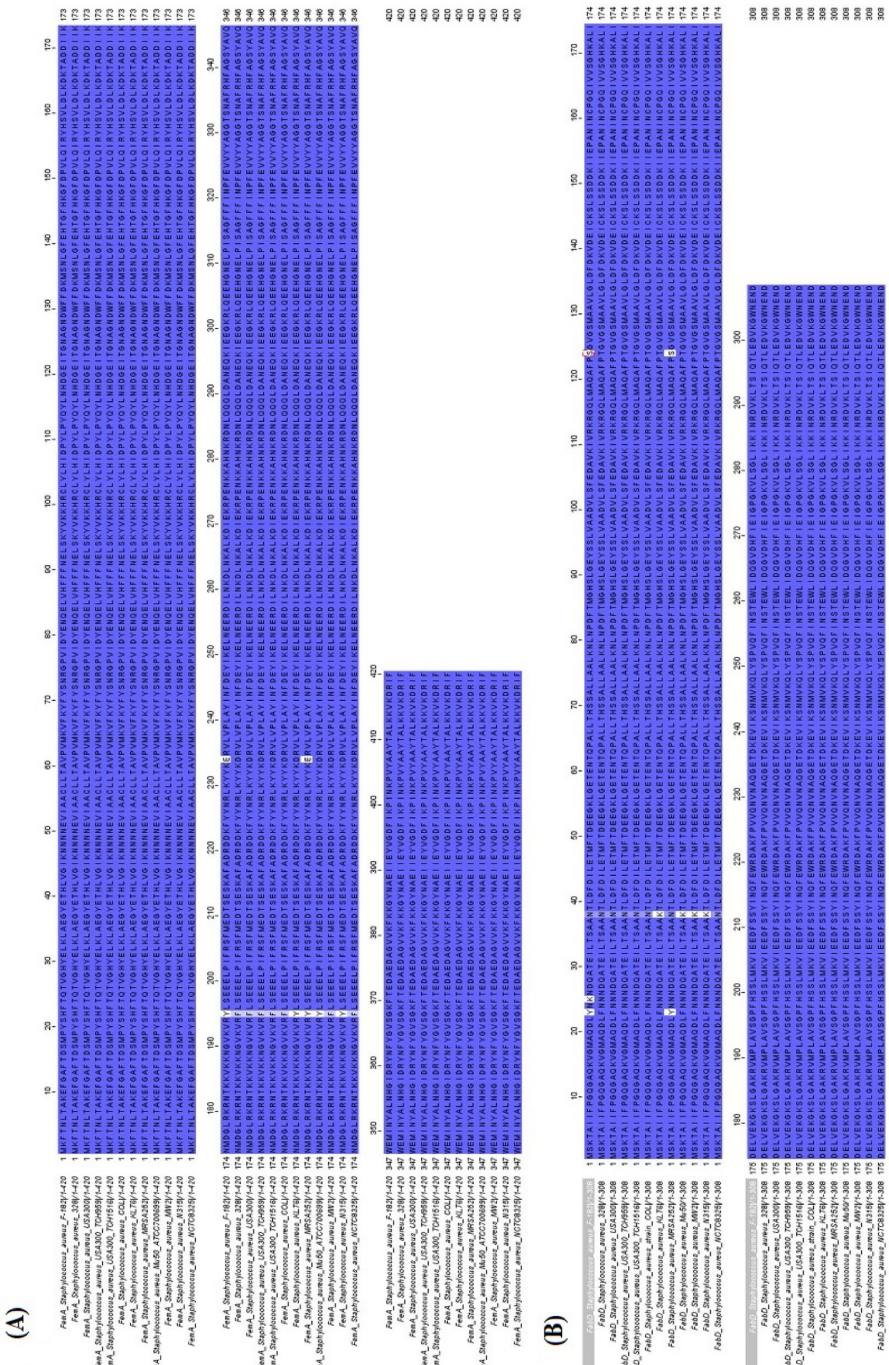
Sequence Alignment Analysis of FemA and FabD Proteins Across MRSA Strains. (A) Multiple sequence alignment of FemA (420 residues) from *Staphylococcus aureus* F-182, 328, and 10 MRSA strains reveals high conservation with variations at position 195 (Phe/Tyr) and position 234 (Asp/Glu). (B) Multiple sequence alignment of FabD (308 residues) shows variations at four positions: position 23 (Phe/Tyr), position 25 (Asn/Lys), position 38 (Asn/Lys), and position 124 (Thr/Ser). Alignments were performed using Clustal Omega

FemA protein alignment across all strains showed conservation in 418/420 residues, with variations only at position 195 (Phe/Tyr) and 234 (Asp/Glu) (Fig. 2A).

FabD alignment showed variations in 4/308 residues across the 12 strains: position 23 (Phe, two strains Tyr), position 25 (Asn, one strain Lys), position 38 (Asn, four strains Lys), and position 124 (Thr, two strains Ser) (Fig. 2B).

### Molecular docking and network pharmacology of rupatadine with FemA protein

**Fig. 3:**
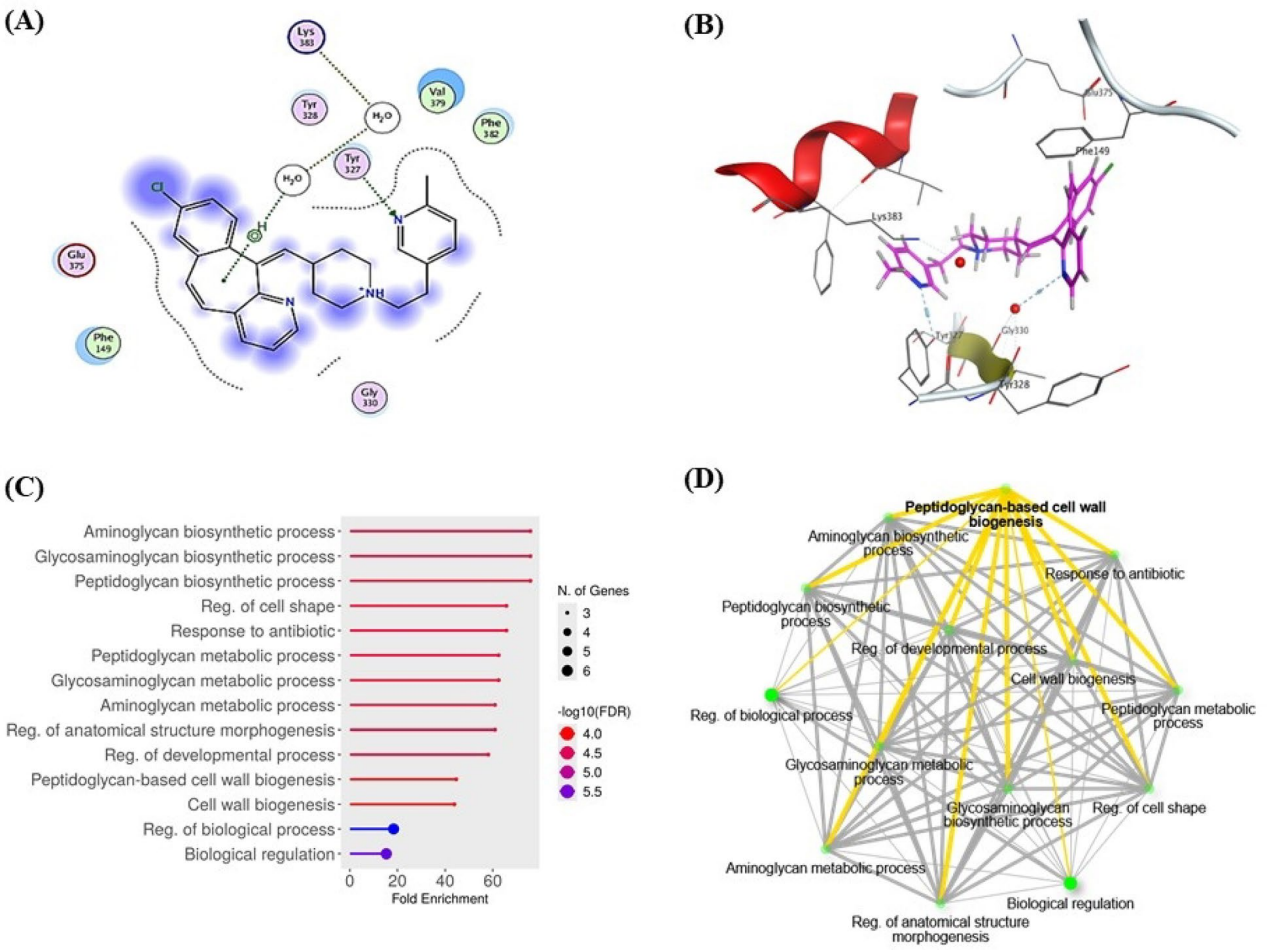
Rupatadine Interacts with FemA and Modulates Peptidoglycan Biosynthesis Pathways in *Staphylococcus aureus*. Two-D Interaction diagram (**A**) and 3D representation (**B**) showing docking pose of rupatadine with the key amino acids in the *S. aureus* FemA protein active site (Distances in Å). Hydrogen bonds between receptor and protein are represented using cyan lines, and amino acids involved in the interaction are labelled. (**C**) Gene Ontology (GO) enrichment dot plot of biological processes affected by predicted protein targets of Rupatadine. Top enriched pathways include peptidoglycan biosynthetic process, response to antibiotic, and cell wall biogenesis, with fold enrichment and statistical significance represented by dot size and color, respectively. (**D**) Pathway interaction network based on GO terms, highlighting peptidoglycan-based cell wall biogenesis as a central node. Yellow edges represent stronger functional linkages among terms, suggesting coordinated modulation of cell wall biosynthesis and related processes by Rupatadine. Darker nodes are more significantly enriched gene sets. Bigger nodes represent larger gene sets. Thicker edges represent more overlapped genes

Rupatadine exhibited a notable binding affinity to FemA, a key enzyme in *S. aureus* peptidoglycan cross-linking, with a docking score of − 3.45 kcal/mol. The docking pose revealed hydrogen bonds with Tyr328 (2.9 Å) and Gly330, along with an arene–H interaction involving Lys383. Additionally, hydrophobic contacts were observed with Phe382, Val379, Arg337, and Phe149 (Fig. 3A, 3B). These interactions suggest a stable and potentially inhibitory binding mode within the active groove of FemA.

To elucidate the broader pharmacological impact of this interaction, a network pharmacology analysis was conducted. Gene Ontology enrichment highlighted significant modulation of biological processes related to peptidoglycan biosynthesis, cellular metabolism, and ultimately peptidoglycan-based cell wall biogenesis (Fig. 3C). The pathway interaction network further supported the hypothesis that Rupatadine interferes with critical nodes in the cell wall assembly machinery, particularly through targeting FemA and its associated interactors such as FemB, FemX, and penicillin-binding proteins (Fig. 3D).

Collectively, these findings suggest that Rupatadine compromises cell wall integrity in *S. aureus* by disrupting peptidoglycan cross-linking and associated biosynthetic pathways, making it a promising repurposing candidate for antimicrobial intervention.

### Safety of rupatadine on skin fibroblasts (HSFs) cell line

**Fig. 4:**
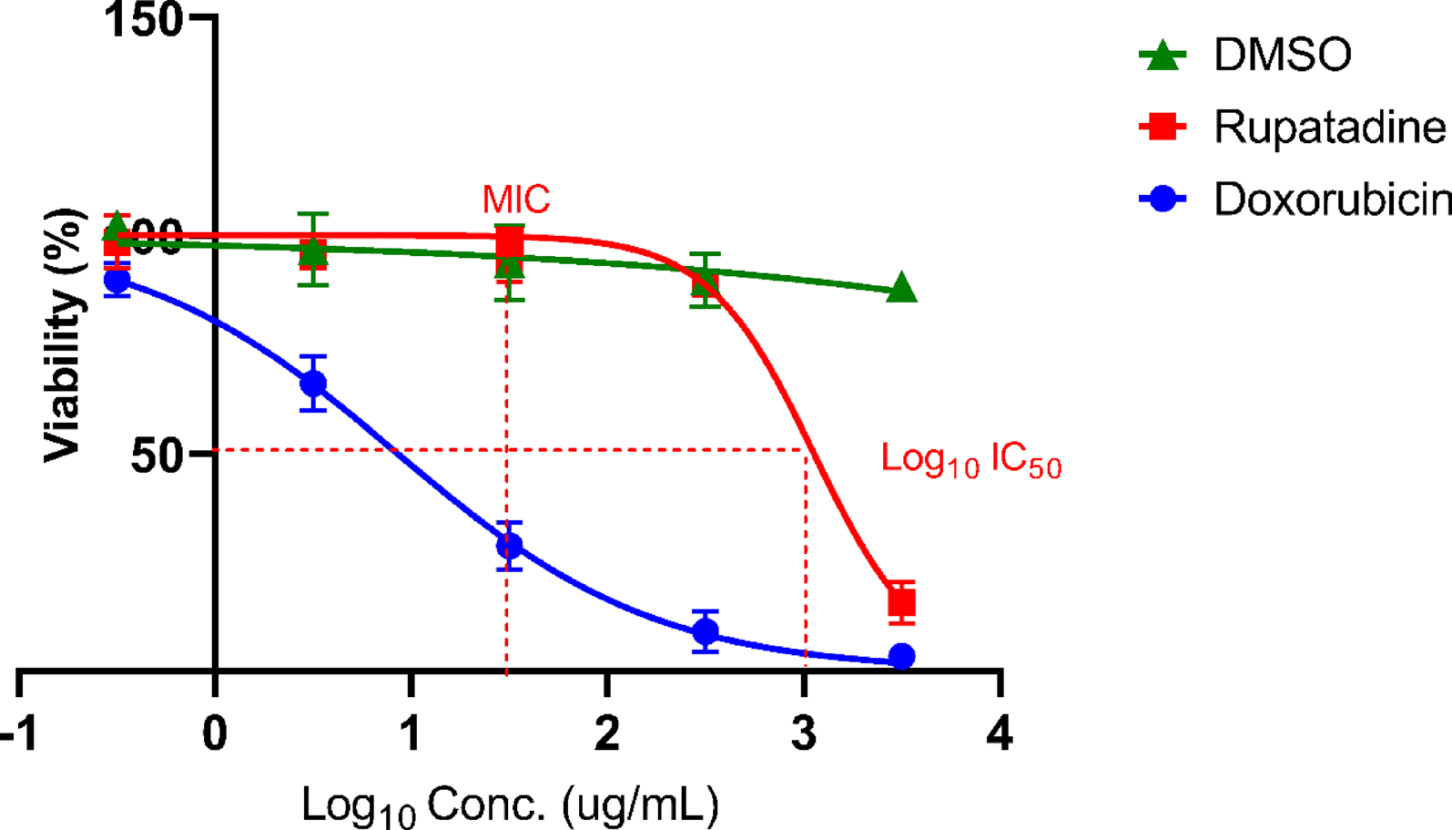
Dose–Response Curves for Cytotoxicity Testing on Human Skin Fibroblasts (HSFs). Dose–response relationships of rupatadine, DMSO (vehicle control), and doxorubicin (positive control) on HSFs cell viability. Cells were treated with increasing concentrations of test compounds for 2 h, and cell viability was assessed using the SRB colorimetric assay. IC_50_ values were calculated using GraphPad Prism software. The dotted line indicates IC_50_ of rupatadine (1150 µg/mL). Rupatadine demonstrated minimal cytotoxicity compared to the potent positive control doxorubicin, while DMSO showed no significant cytotoxic effects across all tested concentrations

Rupatadine showed an IC_50_ of 1150 μg/mL on HSFs, while DMSO (vehicle) maintained > 50% cell viability at all tested concentrations, and Doxorubicin demonstrated IC_50_ of 7.5 µg/mL (Fig. 4).

### Resistance development through serial passage

**Fig. 5:**
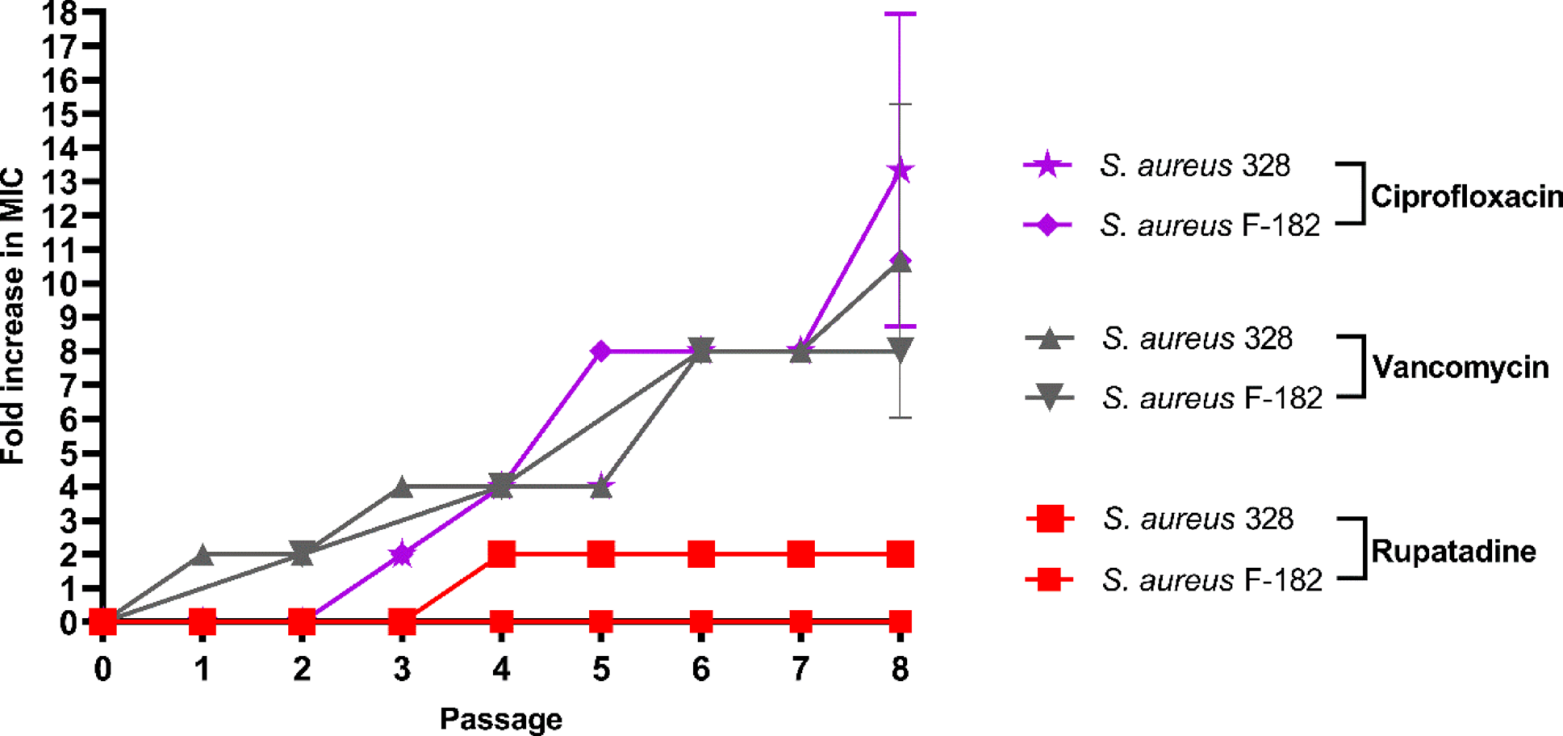
Serial Passage Analysis of Resistance Development in MRSA Strains Treated with Rupatadine, Vancomycin, and Ciprofloxacin. Fold-increase in MIC values during serial passage experiments across eight passages for *Staphylococcus aureus* F-182 and 328. Rupatadine maintained stable MIC values (no increase) with *S. aureus* F-182, and twofold increase in *S. aureus* 328, while vancomycin and ciprofloxacin showed progressive resistance development reaching 8 to 13-fold increases. Data represent mean ± SD of triplicate experiments

Serial passage experiments revealed distinct resistance development patterns among the tested compounds across both MRSA strains (Fig. 5).

Against *S. aureus* F-182, rupatadine demonstrated remarkable stability with no detectable MIC increase throughout eight passages. In contrast, both vancomycin and ciprofloxacin showed progressive resistance development, with vancomycin reaching an eightfold MIC increase and ciprofloxacin achieving a tenfold increase by passage 8.

Against *S. aureus* 328, the resistance development pattern was more pronounced for antibiotics, while rupatadine showed only minimal fluctuation (≤ twofold) that remained within the experiment. Vancomycin showed gradual resistance development reaching an 11-fold MIC increase by passage 8, while ciprofloxacin demonstrated the most dramatic resistance development, achieving a 13-fold MIC increase by passage 8.

### Synergistic effects of rupatadine with conventional antibiotics

Checkerboard assays were conducted to evaluate the combinatorial antimicrobial effects of rupatadine with five conventional antibiotics against both MRSA strains (F-182 and 328). FICI values revealed distinct interaction profiles depending on the antibiotic class and bacterial strain. Synergistic effects (FICI ≤ 0.5) were observed with β-lactam antibiotics, specifically cefazolin and cefotaxime. Against *S. aureus* 328, both cefazolin and cefotaxime demonstrated strong synergism with rupatadine (FICI = 0.3), indicating enhanced antimicrobial activity when used in combination. However, the synergistic pattern was strain-dependent, as *S. aureus* F-182 showed synergism only with cefazolin (FICI = 0.3), while the cefotaxime-rupatadine combination resulted in additive effects (FICI = 0.6).

The aminoglycoside amikacin showed consistent additive effects with rupatadine across both strains (FICI = 0.6). Similarly, linezolid, an oxazolidinone antibiotic typically effective against MRSA, demonstrated additive interactions (FICI = 1.0) for both strains. Vancomycin, the gold standard for MRSA treatment, also showed additive effects (FICI = 1.0) when combined with rupatadine across both strains. No antagonistic interactions (FICI > 2.0) were observed in any of the tested combinations, indicating that rupatadine does not interfere with the antimicrobial activity of conventional antibiotics.

### In vivo efficacy of rupatadine in treating MRSA skin infection in mice

**Fig. 6:**
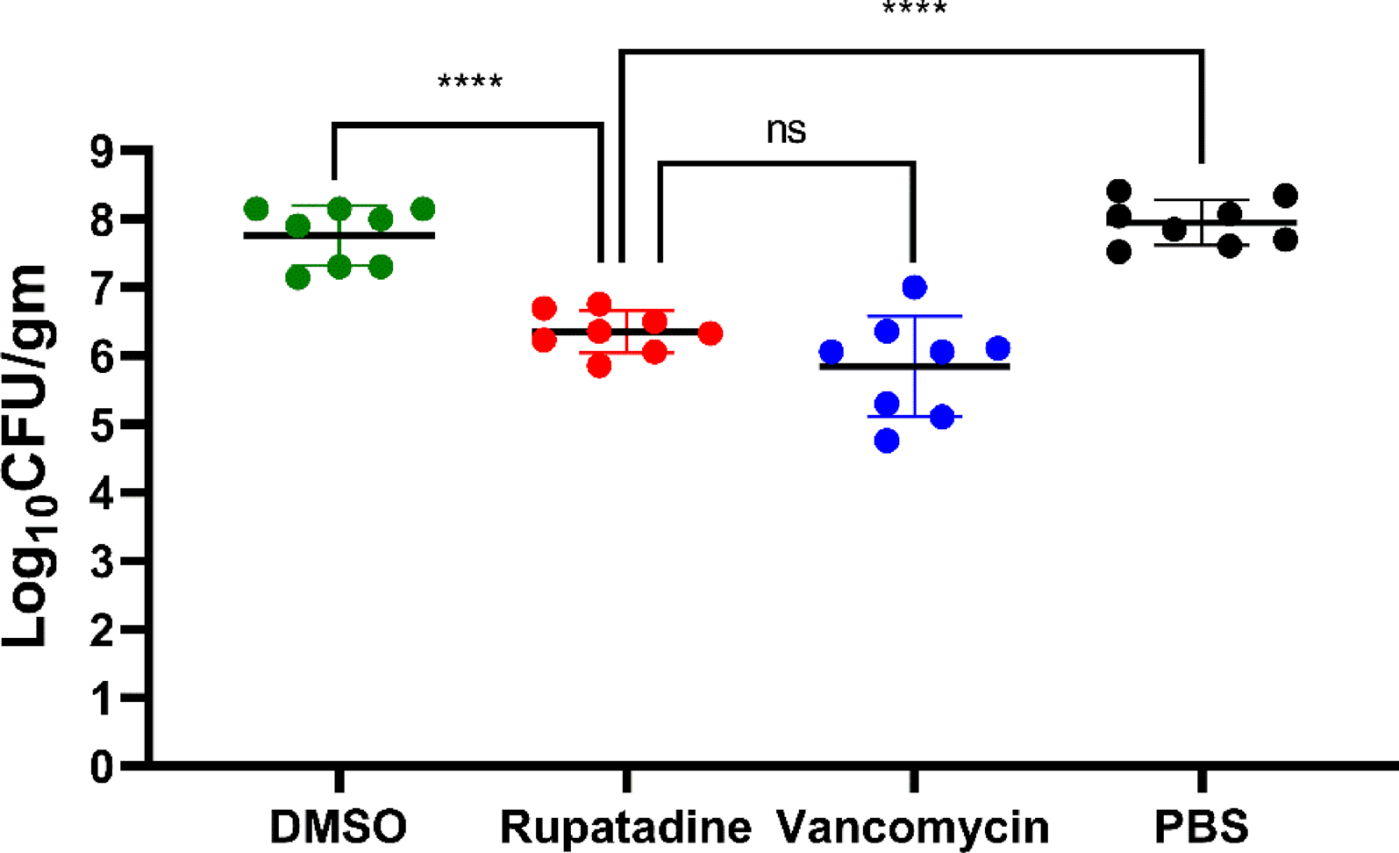
Viable *Staphylococcus aureus* Count in Skin after Topical Treatment with the Tested Agents. Male Swiss mice (n = 8) were subcutaneously injected with S. aureus F-182, followed by five days of topical application of either DMSO, rupatadine, vancomycin, or PBS. Bacterial counts are expressed as colony-forming unit (CFU) per gram of skin. Quadruple asterisk (****) denotes significant values (*p* < 0.0001); ns denotes non-significant value (*p* > 0.05). Data were analyzed using one-way ANOVA (GraphPad Prism 8.4.2)

In a murine MRSA skin infection model (n = 8/group), five-day topical treatment with rupatadine showed comparable bacterial reduction to vancomycin (*p* = 0.1807), with both treatments significantly reducing bacterial counts compared to DMSO and PBS controls (*p* < 0.0001) (Fig. 6).

## Discussion

The emergence and spread of MRSA continue to pose significant challenges in healthcare settings worldwide (Boswihi and Udo 2018; Nandhini et al. 2022). One strategy to solve this fast-paced growing problem is repurposing of existing drugs with known safety profiles, thereby saving time, money, and resources, rather than the conventional way for discovering new antimicrobials (Ibrahim et al. 2020).

While computational methods can identify novel drug targets and potential antimicrobial candidates (Ibrahim et al. 2020), only 10–40% of predictions prove valid experimentally (Sadybekov and Katritch 2023). Though some studies provide both in vitro and in vivo validation (Ibrahim et al. 2020, 2022), many bioinformatic analyses lack experimental confirmation.

This study contributes to the growing body of evidence supporting the use of in silico approaches in identifying and characterizing potential antimicrobial compounds. By corroborating and extending previous findings, we highlight the value of computational methods in guiding future experimental studies and drug development efforts. However, it is important to note that while in silico studies provide valuable insights, further experimental validation is necessary. This study aimed to evaluate potential non-antibiotic compounds with anti-MRSA activity, focusing on repurposing existing drugs to combat this persistent pathogen.

Our analysis of 198 MRSA protein targets (Rahman and Das 2021) initially removed 145 non-druggable proteins, and ended with 10 essential, druggable targets with non-antibiotic ligands. These targets are cytoplasmic, lack human homologs, exist in ≥ 80% of common pathogens, and have available 3D crystallographic structures in PDB.

BLASTP analysis of the 21 selected protein targets against healthy human skin microbiome was conducted to assess potential off-target effects on beneficial bacteria, despite the inherent limitation that highly conserved targets across common pathogens are likely to be similarly conserved in commensal microorganisms (National Research Council 2010b). While this conservation pattern prevents the use of skin microbiome presence as a strict subtractive criterion for target elimination, the analysis can guide future drug development strategies, such as formulation approaches or combination therapies that might minimize adverse effects on beneficial skin microbiome while maintaining antimicrobial efficacy against pathogenic bacteria.

The prevalence of small molecule interactions among identified targets aligns with traditional drug development for bacterial infections. Small molecules offer advantages including better cell membrane penetration, oral bioavailability, and lower production costs (Beck et al. 2022). Drug status analysis revealed that all identified drugs held approved status, suggesting established safety and efficacy profiles. The small fraction of experimental compounds (1.8%) indicates most therapeutic approaches targeting these proteins have progressed beyond early development. Notably, nutraceutical agents (9.8%) suggest potential alternative therapeutic strategies with favorable safety profiles (Gupta and Sharma 2022).

The approach to target essential bacterial processes aligns with recent trends in antibiotic discovery to overcome resistance mechanisms (Ibrahim et al. 2020; Zhang and Lin 2009). Our focus on broad-spectrum targets could potentially address not only MRSA but also other prevalent bacterial infections; an approach previously employed to narrow the search for more prominent targets (Ibrahim et al. 2020; Raman et al. 2008).

The successful prediction of 3D structures for all 21 conserved MRSA protein targets using AlphaFold v2.3.2 provided crucial structural insights for drug discovery efforts. The conservation of these protein targets across ≥ 80% of bacterial pathogens suggests their potential as broad-spectrum antimicrobial targets, making them attractive candidates for developing therapeutics with activity against multiple resistant bacterial species.

The availability of 3D protein structures is crucial for structure-based drug discovery (Kitchen et al. 2004), providing essential information for understanding protein–ligand interactions and conducting reliable molecular docking studies (Liu et al. 2015). Proteins lacking PDB structures, while potentially valuable targets, would require experimental structure determination or high-quality homology modeling before effective utilization in structure-based drug design workflows (Muhammed and Aki‐Yalcin 2019).

From the shortlisted targets, we identified 20 potential binding ligands and selected five diverse candidates: biotin, citric acid, glycine, orlistat, and rupatadine. This approach follows successful research demonstrating non-antibiotic drug repurposing for antibacterial activity, including organic acids (Badie et al. 2022; Ibrahim et al. 2023, 2020, 2022), natural extracts, anti-cancer agents (Kamurai et al. 2020), and anti-depressants (Rácz and Spengler 2023). Screening revealed promising anti-MRSA activity for rupatadine, orlistat, and citric acid.

Citric acid’s antibacterial properties have gained attention recently. Our in silico findings align with previous studies demonstrating its efficacy against pathogens like *Helicobacter pylori* and *Acinetobacter baumannii* (Badie et al. 2022; Ibrahim et al. 2020). Computational approaches have identified glutamine synthetase and riboflavin synthase as key targets, both essential for bacterial survival (Kanehisa and Goto 2000; Zhang and Lin 2009). Citric acid’s anti-MRSA activity (MIC 2.5 mg/mL) highlights its potential as an antibacterial agent, particularly for topical applications (Ormerod et al. 2011), addressing the need for alternatives against resistant pathogens.

Malonyl-CoA acyl carrier protein transacylase (MCAT), crucial for bacterial fatty acid synthesis, is a promising antibacterial target (Rahman and Das 2021; Zhang et al. 2007). Computational studies suggest orlistat may interact with MCAT (Rahman and Das 2021). Kitadokoro et al. identified an additional lipid metabolism target for orlistat (Kitadokoro et al. 2020), not detected in our study or by Rahman and Das (Rahman and Das 2021), potentially because computational approaches often rely on essential gene databases like DEG (Kanehisa and Goto 2000), OGEE (Gurumayum et al. 2021), or BioCyc (Karp et al. 2017)), which may overlook non-essential targets important for bacterial virulence or conditional survival. Nevertheless, orlistat demonstrated experimental anti-MRSA activity with an MIC of 1.5 mg/mL.

Rupatadine, a second-generation histamine antagonist (Merlos et al. 1997), demonstrated remarkable anti-MRSA activity with the lowest MIC (0.03125 mg/mL) against both tested strains.

Its putative target, aminoacyl transferase (FemA), is crucial for peptidoglycan biosynthesis—a well-established target for various antibiotics and non-antibiotic agents (Kanehisa and Goto 2000; Moutiez et al. 2017). The FemXAB family of enzymes is responsible for the sequential building of interpeptide strands in *S. aureus*. Members of this enzyme family represent potential targets for the development of new antibacterial agents. While FemB is not essential for bacterial survival, both FemX and FemA are essential, and their inhibition can lead to bacterial death (Benson et al. 2002).

Our conservation analysis revealed high conservation of FemA across multiple MRSA strains, supporting its status as an essential gene in *S. aureus* (Hürlimann-Dalel et al. 1992). This high conservation suggests rupatadine’s effectiveness might extend across various MRSA strains. Similarly, FabD (MCAT), orlistat’s target, also showed high conservation across MRSA strains. Targeting such highly conserved proteins is valuable for drug development, potentially reducing the likelihood of rapid resistance development while ensuring efficacy across different pathogen strains.

Rupatadine’s antibacterial activity aligns with growing evidence supporting antimicrobial properties of antihistamines. El-Nakeeb et al. demonstrated antimicrobial activity of several antihistamines (azelastine, cyproheptadine, mequitazine, and promethazine) against various pathogens including *S. aureus*, with MICs ranging from 62.5 to > 1000 µg/mL (El-Nakeeb et al. 2011). Rupatadine’s antimicrobial spectrum extends beyond MRSA, with demonstrated efficacy against various Mycobacteria strains (MICs: 3.13–12.5 µg/mL) (Tian et al. 2024). Other antihistamines like terfenadine and cetirizine dihydrochloride have shown similar activity (Maji et al. 2017; Perlmutter et al. 2014).

Given rupatadine’s superior performance, we conducted molecular docking simulations to investigate its binding mechanism with FemA, an approach successfully used in numerous studies to identify potential antibacterial compounds (Badie et al. 2022; Ibrahim et al. 2023, 2022; Rahman and Das 2021).

The network pharmacology approach successfully predicted FemA as a primary molecular target for Rupatadine, and the molecular docking showed favorable binding affinity (− 3.45 kcal/mol) indicating effective engagement with this critical enzyme in bacterial cell wall biosynthesis (Benson et al. 2002). The molecular docking analysis revealed stable complex formation through hydrogen bonding with Tyr328 and Gly330, arene-H interaction with Lys383, and hydrophobic contacts with Phe382, Val379, Arg337, and Phe149. These interactions within the active groove suggest direct interference with FemA’s catalytic function through competitive or allosteric mechanisms. While these in silico results provide valuable insights, they represent a simplified model of the biological interaction. The favorable binding energy should be considered alongside rupatadine’s potent in vitro activity against MRSA, suggesting factors beyond simple binding affinity (cellular penetration, secondary targets) may contribute to its effectiveness.

Gene Ontology enrichment analysis demonstrated that Rupatadine’s antimicrobial effect extends beyond simple enzyme inhibition to encompass systemic disruption of peptidoglycan biosynthesis pathways. The pathway interaction network revealed FemA as a central hub interacting with critical enzymes including FemB, FemX, and penicillin-binding proteins, suggesting that FemA inhibition could have cascading effects throughout the cell wall assembly pathway, potentially amplifying antimicrobial impact beyond single-enzyme targeting.

While computational predictions provide strong evidence for antimicrobial potential, experimental biochemical validation remains essential for confirming these findings and supporting further development of Rupatadine as a repurposed antimicrobial agent targeting *S. aureus* cell wall biosynthesis.

Since rupatadine’s introduction to the markets in 2003, the drug has been considered safe and well-tolerated in adults and children over 2 years old, with no specific warnings or pharmacovigilance alerts issued (González-Núñez et al. 2016). This established safety profile is a significant advantage of drug repurposing, as the documented safety of already approved drugs reduces potential risks in new applications (Ibrahim et al. 2020). Our in vitro cytotoxicity assay on HSFs further supports rupatadine’s safety profile, revealing a relatively high IC_50_ of 1150 μg/mL. This value, being significantly higher than the MIC (*p* < 0.0001), suggests a favorable therapeutic window for topical application. The substantial difference between the cytotoxic concentration and the effective antimicrobial concentration indicates that rupatadine could potentially be used at therapeutically effective doses with minimal risk of toxicity to human cells. This safety evaluation is crucial for the subsequent in vivo assessment of the drug’s efficacy, as it provides a foundation for determining appropriate dosing regimens and application methods in future clinical studies.

The serial passage experiments provide crucial insights into the long-term therapeutic potential of rupatadine compared to established antibiotics. The complete absence of detectable resistance development against rupatadine across eight passages and two different MRSA strains represents a significant advantage over conventional antibiotics. This finding is consistent with similar observations reported for non-antibiotic repurposed compounds against *H. pylori*, where drug repurposing candidates have demonstrated absence of resistance development compared to traditional antimicrobial agents, even after 14 passages (Ibrahim et al. 2023, 2022). This is particularly noteworthy given that both vancomycin and ciprofloxacin, which are clinically important antibiotics, showed substantial resistance development under identical experimental conditions. The rapid resistance development observed with ciprofloxacin (up to 13-fold MIC increase) aligns with long well-documented clinical resistance patterns for fluoroquinolones against *S. aureus* (Blumberg et al. 1991). Similarly, the gradual but sustained resistance development against vancomycin reflects emerging clinical concerns about vancomycin-intermediate *S. aureus* strains (Howden et al. 2014). The differential resistance development between the two MRSA strains suggests strain-specific factors that may influence resistance acquisition rates, with *S. aureus* 328 showing generally higher resistance development potential.

From a clinical perspective, the slower resistance development of rupatadine suggest it could serve as a valuable long-term therapeutic option, particularly in combination therapies where its resistance stability could help preserve the efficacy of partner antibiotics. The checkerboard assay results reveal promising therapeutic opportunities for rupatadine as an adjunctive antimicrobial agent, particularly in combination with β-lactam antibiotics. The synergistic interactions observed between rupatadine and cefazolin/cefotaxime (FICI = 0.3) are clinically significant, as these combinations could potentially restore the efficacy of β-lactam antibiotics against MRSA strains that are typically resistant to this drug class (Lade and Kim 2023). This synergism suggests that rupatadine may disrupt bacterial resistance mechanisms or target complementary pathways that enhance β-lactam penetration or activity. The combination approach could potentially reduce the required concentrations of both agents, thereby minimizing toxicity while maximizing efficacy. The strain-specific differences in synergistic patterns highlight the importance of strain characterization in combination therapy approaches. These variations may reflect differences in resistance gene expression, cell wall composition, or efflux pump activity between the strains. The consistent additive effects observed with amikacin, linezolid, and vancomycin, while not synergistic, are still therapeutically valuable as they indicate enhanced antimicrobial activity without antagonism.

The in vivo efficacy study of topical rupatadine treatment on MRSA skin infections in a murine model yielded promising results. The five-day topical application regimen demonstrated that rupatadine significantly reduced the viable bacterial count in infected skin compared to both vehicle (DMSO) and untreated (PBS) control groups (*p* < 0.0001 for both). This marked reduction in bacterial load highlights rupatadine’s potential as an effective topical antimicrobial agent against MRSA infections. Notably, the efficacy of rupatadine was comparable to that of vancomycin, a well-established antibiotic commonly used to treat MRSA infections (Brown et al. 2021). The lack of significant difference between the rupatadine and vancomycin groups (*p* = 0.1807) suggests that rupatadine’s antimicrobial activity in vivo approaches that of a standard antibiotic. This finding is particularly significant given the growing concern over vancomycin-resistant strains (Shariati et al. 2020), and also particularly encouraging, considering rupatadine’s current use as an antihistamine and its well-documented safety profile combined with its safety on tested cell lines in this study. The ability of rupatadine to achieve such significant bacterial reduction in a topical application further supports its potential for repurposing as a skin infection treatment.

In conclusion, drug repurposing represents a promising option in combatting antibiotic resistance crisis. Rupatadine demonstrated significant anti-MRSA efficacy both in vitro and in vivo, with computational approaches successfully predicting this candidate to interfere with FemA protein target. Given rupatadine’s established safety profile, it presents a viable repurposing opportunity for MRSA treatment. While our in vitro and murine models demonstrated promising anti-MRSA activity of rupatadine, we recognize the limitation of these systems to fully replicate the complexity of human infections. This warrants further clinical investigation to validate these findings.

## Supplementary Information

Below is the link to the electronic supplementary material.

Supplementary Material 1

Supplementary Material 2

Supplementary Material 3

## Data Availability

Information on binding ligands was extracted from DrugBank repository: https://www.drugbank.ca. Sequence alignment for similarity check in common pathogens and genetic relationships between the tested strains were performed using NCBI BLAST suite: https://blast.ncbi.nlm.nih.gov/Blast.cgi. Prediction of 3D structure of MRSA’s protein targets was done using AlphaFold v2.3.2: https://github.com/google-deepmind/alphafold. The visualization fo the predicted 3D protein structures was done using Mol* Viewer: https://molstar.org/. Proteins ID were collected from UniProt database: http://www.uniprot.org/ . Information on protein 3D structures was extracted from Protein Data Bank repository: https://www.rcsb.org. Multiple sequence alignment and homology analysis were performed using Clustal Omega: https://www.ebi.ac.uk/Tools/msa/clustalo/. Target prediction, enrichment analysis, and network construction were performed using the databases: SwissTargetPrediction: http://www.swisstargetprediction.ch/; PharmMapper: http://lilab-ecust.cn/pharmmapper/index.html; DAVID: https://david.ncifcrf.gov/; KEGG: http://www.kegg.jp/; and ShingGo v0.81: https://bioinformatics.sdstate.edu/go/.

## Abbreviations

3D: Three-dimensional
CLSI: Clinical and Laboratory Standards Institute
DAVID: Database for annotation, visualization, and integrated discovery
DMSO: Dimethyl sulfoxide
FemA: Aminoacyl transferase
FICI: Fractional inhibitory concentration index
GO: Gene ontology
HSFs: Human skin fibroblasts
IACUC: Institutional animal care and use committee
IC_50_: Half-maximal inhibitory concentration
KEGG: Kyoto encyclopedia for genes and genomes
MBCs: Minimum bactericidal concentrations
MCAT: Malonyl-CoA acyl carrier protein transacylase
MIC: Minimum inhibitory concentration
MRSA: Methicillin-resistant *Staphylococcus aureus*
MSA: Mannitol salt agar
SRB: Sulforhodamine B
PBS: Phosphate buffer saline
PDB: Protein data bank
